# Polygenic risk scores and embryonic screening: considerations for regulation

**DOI:** 10.1136/jme-2024-110145

**Published:** 2024-12-16

**Authors:** Casey M Haining, Julian Savulescu, Louise Keogh, G Owen Schaefer

**Affiliations:** 1 Centre for Health Equity, Melbourne School of Population and Global Health, The University of Melbourne, Melbourne, Victoria, Australia; 2 Centre for Biomedical Ethics, Yong Loo Lin School of Medicine, National University of Singapore, Singapore; 3 Uehiro Oxford Institute, University of Oxford, Oxford, UK

**Keywords:** Reproductive Medicine, Ethics- Medical, Policy

## Abstract

Polygenic risk scores (PRSs) have recently been used to inform reproductive decision-making in the context of embryonic screening. While this is yet to be widespread, it is contested and raises several challenges. This article provides an overview of some of the ethical considerations that arise with using PRSs for embryo screening and offers a series of regulatory considerations for jurisdictions that may wish to permit this in the future. These regulatory considerations cover possible regulators and regulatory tools, eligibility criteria, information and education requirements and the need for ongoing refinement of the relevant technology, research and consultation.

## Introduction

 Polygenic risk scores (PRSs) can provide a quantitative metric of an individual’s predisposition to developing polygenic diseases (eg, diabetes, hypertension, breast cancer and coronary artery disease) or insight into non-disease parameters (eg, intelligence, height, criminality, same-sex behaviour and endurance) based on the cumulative effect of gene variants derived from genome-wide association studies (GWAS) using single-nucleotide polymorphisms.^1^,^5^ These studies draw on large cohorts of genotyped individuals where phenotypes for certain quantitative or dichotomous traits (cases and controls) are known.^6^

While PRSs can be useful at the population level, they are less useful at predicting disease susceptibility at the individual level and, therefore, can only be used as screening tools rather than diagnostically.^7^ Current technology also has limited specificity and sensitivity.^1^ It cannot provide insight into the potential severity of conditions, which limits the predictability of PRSs in the context of polygenic conditions given their highly variable presentation in terms of severity and chronicity.^8 9^ PRSs are also inherently dependent on the rigour and comprehensiveness of the GWAS used to calculate them and hence have poor portability.^7 10^ Indeed, PRSs will be most predictive in populations whose characteristics match those who participated in the GWAS they were calculated from, which currently only test for a small number of diseases and predominately comprise those of European ancestry.^11 12^ ^2^ Similarly, the predictive accuracy of PRSs will vary depending on a person’s sex, age and socioeconomic status.^9^ The limited predictive ability of PRSs are also reflected in the fact that they cannot fully account for pleiotropy (ie, the tendency for genetic variants to affect multiple phenotypes), nor can they account for environmental factors.^11 13^

Despite these limitations, PRSs have been used clinically to inform population screening programmes, refine risk for individuals undergoing genetic testing for monogenic risk genes, guide therapeutic interventions, facilitate diagnosis and predict health outcomes.^10^ This article, however, is concerned with a more recent use of PRSs, namely their use in the reproductive context for embryo screening as part of preimplantation genetic testing (PGT).

PGT is the process that tests embryos during the in vitro fertilisation (IVF) process for hereditary genetic disorders and chromosomal abnormalities to prevent the birth of a child with a hereditary condition or increase the IVF success rate. PGT has predominately been limited to screening for monogenic conditions, structural arrangements and aneuploidy. More recently, however, PRSs have been used to screen embryos, sometimes called PGT-P or polygenic embryo screening (PES).^3^ Like other forms of PGT, this form of PGT is intended to inform prospective parents’ decisions on which embryo to transfer. However, in the context of PGT-P, the calculation of PRSs enable prospective parents to compare the risk of embryos developing polygenic conditions or traits.^9 14^

The use of PRSs in the context of embryo screening raises several ethical, social and legal concerns. There is currently limited evidence that supports the efficacy of PRSs in embryo screening, but a few studies demonstrate promise for this technology.^15^,^17^ Critics, however, have argued that this evidence should be interpreted cautiously, given these studies are carried out by researchers with commercial interests.^8^ While studies among professionals demonstrate more scepticism about using PRSs for embryo screening,^18 19^ surveys among the broader public and IVF users show greater support for such a use.^19^,^21^ Notably, however, most studies exploring attitudes have been carried out in the US and have generally shown greater support compared with other cultural contexts (eg, Europe).^22^

In practice, the use of PRSs for embryo screening has been limited to date, with most reports stemming from the US, although there has also been a reported case of embryo selection using PRSs in China.^23^ In the context of the US, companies such as Orchid Biosciences and Genomic Prediction offer to calculate PRSs for embryo screening.^24 25^ The number of couples who have accessed PRSs for embryo screening from Genomic Prediction has been reported to be in the low hundreds.^26^ While these companies can offer PRSs in relation to disease and non-disease traits, the availability of the latter has become more limited. Indeed, Genomic Prediction initially marketed that it could screen for intellectual disability but has since retreated from offering this service. Justifying its position, Genomic Prediction’s founder, Stephen Hsu, stated, ‘traits like height and cognitive ability are too controversial and detract from our ability to help families reduce disease risk’.^27^ Limiting the availability to disease traits also reflects public views, which show greater support for using PRSs for disease traits and less support for non-disease traits.^21^

Little is known about the desire of other countries to permit PRSs to be used in the context of embryonic screening. However, if PRSs are used in this way, then appropriate consideration of the ethical implications and potential regulatory frameworks is salient. To this end, this article explores some ethical concerns with implementing PRSs in the context of embryonic screening before offering a series of regulatory considerations in light of such concerns.

## Ethical considerations

Several ethical considerations arise in the context of PRSs used for embryonic selection. In this section, we aim to canvass some of the ethical arguments raised in this context. While a myriad of ethical considerations could be discussed, we have confined our discussion to four broad classes of ethical arguments that we deem to be most relevant, including (a) reproductive autonomy, (b) the harm principle, (c) eugenics, discrimination and designer babies and (d) justice. In doing so, we do not purport to have covered all the ethical issues and acknowledge that some of the arguments we advance may have relevance to multiple ethical frameworks.

### Reproductive autonomy

Respect for reproductive autonomy is perhaps the most prominent argument in favour of using PRSs for embryo screening. This principle states that prospective parents should be able to make their own informed reproductive choices, which extends to reproductive screening and treatment options.^28^ Such an argument stems from a need to protect parental choice and minimise paternalism. While on its face, the availability of PRSs to preference particular embryos may be construed as advancing reproductive autonomy by offering more information, some commentators are critical about the extent to which this can be realised. Indeed, arguably, marketing the use of PRSs for embryo screening to advance reproductive autonomy is somewhat of a façade due to societal pressure. As Rativsky has argued, in hypercompetitive societies, the autonomy of choice is an ‘illusion’ as people often feel societal pressure to use ‘add-on services’ (such as screening and testing services during IVF) and, therefore, may feel compelled to generate a PRS and use this to inform their reproductive choices, despite not necessarily desiring to.^27^

Furthermore, given the epistemic limitations discussed above, PRSs may provide low-quality information that could mislead and thereby inhibit autonomy rather than promote it. Indeed, while calculating PRSs for embryos does provide further information to inform decision-making, there is no guarantee this will provide prospective parents with an ‘obvious choice’ when it comes to selecting embryos.^4^ When faced with the choice of comparing scores, prospective parents will be limited in selecting embryos that are genetic siblings, which ultimately provides a limited gene pool.^12^ Although this preserves genetic relationships (because you are selecting between sibling embryos), the difference between PRSs calculated among sibling embryos will be substantially less than random embryos in the general population, limiting the potential benefits of this technology.

Further, even among this limited gene pool, prospective parents will still face difficult decisions, especially when required to select between otherwise unequal variables when screening multiple polygenic conditions simultaneously.^8^ Consequently, it may be the case that when selecting for one trait, parents may inadvertently be selecting against others, including ones that could potentially be desirable to them and the future child (eg, selecting for educational attainment has been found to increase the absolute risk for bipolar disorder).^11^ Polyakov *et al* cogently demonstrate this point by providing an example of a choice parents may face, specifically describing the difficulty between choosing a future child with a 5% risk of developing type 2 diabetes by age 50 and one that has a 10% risk of developing Alzheimer’s disease by age 80.^2^ While this poses challenges, there is evidence suggesting that many diseases have a phenotypic correlation; therefore, selecting against one disease may simultaneously reduce the risk of many diseases.^29^

Furthermore, given that PRSs are calculated from GWAS, they cannot account for the influence of environmental factors and how this may impact the phenotype of the future child.^11^ This is particularly limiting given that many polygenic conditions are adult-onset; hence, the environment’s impact on the phenotype may be considerable. Indeed, the delay between diagnosis and onset may mean that effective treatment and preventative measures (including new treatment options resulting from research and technological innovations emerging postdiagnosis) may influence the adult phenotype^2 30^ This inadvertently impacts the decision-making process. Using the above example concerning Alzheimer’s disease, even if the parents judge Alzheimer’s disease to be the more devastating disease today, the decades of research and development until the child turns 80 could render it much more manageable compared with diabetes.

Due to the many factors that can ultimately influence a phenotype, there is a need to balance several variables to select the ‘right’ embryo, particularly in light of the abovementioned difficulties. This could arguably create a fallacy of ‘control’ over reproduction and a sense of parental responsibility for the traits of a child. Similarly, concerns have been raised in relation to prospective parents being faced with information overload and potential decision fatigue.^8 31^ This may, in turn, result in a decision-making process that is highly emotional for patients and may even create anxiety around making the ‘right’ decision or indeed a decision at all.^9 18^

### Harm principle

Given reproductive choices are largely a private matter, the degree to which the state can interfere is often considered through the lens of the harm principle, particularly in Western democracies. Adopting a libertarian view and drawing on John Stuart Mill’s harm principle, restricting the accessibility of PRSs for embryo selection would only be justified based on preventing harm to other parties or, as Saunders has argued, preventing harm to non-consenting parties.^32^ In this case, the most relevant party other than the consenting prospective parents would be the future child.

The risks and harms associated with IVF and PGT are well documented, including physical risks associated with IVF both to the prospective parent undergoing IVF as well as the child, such as pre-eclampsia, abnormal placentation, low birth weight, prematurity and miscarriage,^7^ as well as psychological harms, including misshapen parental expectations. Notably, these are not the risks of PRSs as such, at least when IVF would be undertaken even without the availability of PRSs (as is the case with present applications). Instead, the harm principle would focus on the marginal harm of generating and providing a PRS to prospective parents.

Currently, the calculation of PRSs is marketed as an add-on to current IVF treatments. Accordingly, limited additional risk and harm to the child arise from calculating a PRS if the prospective parents are already undergoing IVF and PGT on another basis (eg, due to infertility or aneuploidy), which we define as ‘supplementary PGT-PRS’.^5^ We note, however, in the case of supplementary PGT-PRS, there may be some additional psychological harms to the prospective parents associated with decision-making (eg, information overload, decision fatigue and the fallacy of choice discussed above) and potential harms associated with having false expectations about the future child (which, in turn, translate to harms for the future child in the form of misshapen parental expectations).^9 21^

In cases where IVF (or multiple cycles of IVF) is sought merely to have a PRS calculated and select an embryo (which we define as ‘non-supplementary PGT-PRS’), the potential harms to parties is more significant. First, the harms and risks associated with IVF discussed above would not have been sustained but for the desire to calculate a PRS.^2^ Moreover, parents may undertake multiple IVF cycles to look for a supposedly ideal embryo, which will result in more embryos generated and potentially a greater number discarded.^18^ Most importantly, if PGT poses risks to the embryo, this may manifest as harm to a future child, which is the strongest reason to restrict liberty.^33^ The ethical issues are thus very different for non-supplementary PGT-PRS compared with supplementary PGT-PRS.

### Eugenics, discrimination and designer babies

Eugenic concerns have frequently been raised in light of advances in reproductive technologies (such as the advent of PGT), which have resulted in more information being available to guide reproductive decision-making and greater opportunities for selection.^26^ Such eugenic concerns acknowledge the possibility that this technology has the potential to create designer babies and a ‘Gattaca-type world’.^26 34 35^ Without proper public engagement and oversight, there is potential for the practice of calculating PRSs to lead to increased stigmatisation and discrimination of certain conditions.^12^ Indeed, judgements about which traits are considered desirable or undesirable are often informed by prejudicial values, including racist, sexist and ableist ones.^35^ The ability to select also provides those already better off with a competitive advantage and has been argued to exacerbate social inequality.^20 23^ Arguably, such a risk is heightened in the case of non-disease traits. Indeed, some argue that using PRSs for non-disease traits such as educational attainment and income echoes historical eugenic policies, where attempts were made to eliminate undesirable traits from the gene pool.^2 8^ This, in turn, incites fears about reducing the population’s overall diversity and individualising blame for genetic traits.

Relatedly, concerns about the use of PRSs in the reproductive context re-enliven debates about designer babies, whereby, *inter alia*, concerns around consumerism and the commodification of children arise. Notably, however, using PRSs for embryo screening differs from genome editing with CRISPR-Cas9 technology, given the former does not result in genetic manipulation or modification.^23 36^ Conversely, using PRSs for reproductive decision-making is arguably akin to choosing a ‘winning ticket’ in the ‘genetic lottery’ for optimal traits (as defined by the prospective parents) among the various possible combinations and permutations of genes that can occur during fertilisation.^36^

Some commentators endorse the use of PRSs in this context on the grounds of procreative beneficence (ie, the ethical obligation placed on individuals who are provided with the opportunity to select a child) and that this represents a more liberal form of eugenics where parents are responsible for decision-making as opposed to the state.^37 38^ According to this argument, if there is scope to select a child, then we are expected to do so in a way that ensures they have the best life (or at least as good of a life as others) based on available information.^38^ This framework has been argued to support the disclosure of PRSs (to the extent that PRSs are reliable indicators of future well-being) so prospective parents are provided with as much information as possible, which is conveyed in a way in which they can understand to allow them to act in the prospective child’s best interests.^2^ Notably, selection, even under the guise of procreative beneficence, can create high expectations of the child and disrupt the parent–child relationship.^37 39^

### Justice

Distributive justice and related utilitarian arguments also become relevant when introducing any new policy. Most apparent are the costs associated with accessing this screening option and how funding (if any) is allocated. To justify resource allocation using public funds, it must be cost-effective.^37^ Accordingly, there should be endorsement that implementing this technology will result in meaningful benefits to the population compared with if it had not been implemented. However, this is a hard case to make at present, given the limitations of the technology discussed previously.

Moreover, it is likely that PRSs in the reproductive context, at least in their primitive stages, will only be available to couples who can afford IVF. For many countries, even with a comprehensive national healthcare programme, IVF is primarily a privately funded medical service, or at least started as one. Therefore, PES will not likely be widely accessible.^40^ Even if IVF is provided in the public system, it is often limited (eg, number of cycles). Hence, ‘add-on’ services such as PRSs are unlikely to be subsidised (or at least in the foreseeable future), and the availability of subsidy (if any at all) is likely to differ depending on whether a person is accessing supplementary or non-supplementary PRS (with the former more likely to attract subsidy).

Consequently, the use of PRSs will likely only be a viable option for those who can afford to access it.^37 40^ However, if the use of PRSs continues to be confined to the private market, then the cost is borne by parents and issues of justice based on the allocation of limited public resources will not arise. Some may argue that this creates inequality, but if the evidence for public funding is wanting and effectiveness is in question, it is not clear whether this kind of inequality should be rectified by state subsidy. Indeed, if calculating PRSs for embryo screening was precluded or restricted based on the fact that equitable access is unlikely to be achieved, this would be an unacceptable form of levelling down, making some parties worse off and no party better off to try and achieve greater formal equality.^41^

Notwithstanding this, it is not uncommon that when innovative technologies are launched, they are done so on a small scale, and over time, as acceptance builds across society, opportunities for access widen.^40^ Similarly, commentators have argued that while the economic barrier to treatment is a worthy consideration in terms of implementing the technology, the prospect of inequitable access is essentially relevant to any field of medicine or sociotechnical progress, not just this one.^35^

In a different vein, contemplating allocating resources to this technology may seem paradoxical, especially in the absence of universal health coverage that could help prevent many polygenic diseases in the first place. Indeed, it has been argued that de-emphasising the environmental and social determinants of common diseases shifts the public attention away from structural solutions to health and disability challenges towards individual responsibility.^42^

## Regulatory considerations

Having considered the nature of PRSs and their epistemic limitations, as well as some of the ethical arguments for and against their use in the reproductive context, this section aims to build on the preceding discussion and offer some considerations for regulation. To preface our discussion, we acknowledge that there is yet to be widespread support for using PRSs in the context of embryonic screening, especially outside the research setting.^2^ Despite current concerns, it is possible that the technology and the genomic data relied on to calculate PRSs will improve, which could, in turn, enhance the predictive power of PRSs and, hence, their appeal.^11^ Further, it has been argued that there is likely to be increasing market pressures for the clinical costs of IVF and PRSs to fall over time, leading to more widespread use.^11^ Even if such technologies remain unreliable, they may still come into use, particularly in the direct-to-consumer (DTC) sector, if companies aggressively market and there is a consumer demand. Companies may begin to offer PRSs under the belief that the technology is likely to become more widespread or as a result of technological imperatives which create a drive to use the technology merely because it is new.^4^ However, without specific regulatory frameworks, such market pressures lend themselves to a laissez-faire approach to regulation, with the only form of regulation carried out by commercial institutions, which may lack ethical scruples.

The following discussion offers some insights into how the use of PRSs in the context of reproduction can be regulated. In doing so, we acknowledge that approaches to regulation are ultimately driven by context and sociocultural norms. Hence, we do not aim to advance a particular regulatory approach in this section. We also note that given some of the current limitations of PRSs and ethical concerns, not all jurisdictions may desire to implement this technology. However, even in this case, considering regulatory approaches may still be useful and help inform future deliberations should jurisdictions revisit such a stance. Finally, our discussion that follows assumes that a laissez-faire approach to regulation is somewhat undesirable; however, we also concede that restrictive regulatory regimes may invite medical tourism.^43^ With this context in mind, we set out a series of regulatory considerations for policymakers below. Before doing so, we provide some examples of how PGT is currently regulated to provide context for our subsequent discussion of regulatory considerations.

### Current approaches to regulating PGT

Current regulatory approaches to governing PGT are not necessarily apt to deal with the use of PRSs, particularly because they do not explicitly target PRSs (although they could be adapted to do so in the future). Some jurisdictions adopt permissive approaches to regulating PGT, employing a libertarian and private ordering (or bottom-up) approach to regulation.^37 44^ For instance, in the US, where PRSs are being used in this context, there is no overarching legislation nor a federal actor or agency charged with the responsibility of regulating the practice (outside of consumer protections afforded by the state). While some professional guidance does exist, this has been described as ‘scant and insufficient’.^45^ Such guidance tends to be more educational in nature and fails to set out specific requirements, inviting clinician discretion.^45^ This reflects the nature of medicine in the US, which is mainly market-driven and offers leeway to physicians to offer services they want and charge fees they deem appropriate.^44 45^

This permissive approach, however, is not widely adopted globally. In most cases, regulatory approaches to PGT rely on top-down regulation by the state, which is often tight and limited.^46^ Typically, these models operate on what has previously been described as a ‘disease model’, whereby PGT is only permitted to select against (but not for) embryos with particular disease traits rather than non-disease traits.^37^ The frameworks setting out such boundaries often exist in law but may also (or alternatively) feature in guidelines or policy.

The nature of the ‘disease model’ and the conditions under which PGT is permitted varies across jurisdictions. For the purposes of illustration (rather than purporting to be exhaustive or capture all jurisdictional nuances), we provide an overview of regulatory approaches to PGT employed by three countries that broadly adopt three different approaches and highlight some notable differences in their approaches. However, we note that, at present, these jurisdictions do not expressly endorse PRSs for PGT under their existing frameworks.^43 47^

Our first example of a PGT model is one where PGT use is left to the discretion of individual clinicians or providers, referred to by some as the ‘clinical assessments model’.^43^ Australia is one example of a country that largely adopts such a model. In Australia, despite the existence of legislation in some jurisdictions (and associated jurisdictional variation), regulation of PGT in most Australian jurisdictions predominately relies on ethical guidelines issued by the National Health Medical Research Council (NHMRC), compliance with which is required by the regulator, the Reproductive Technology Accreditation Committee.^48^ The guidelines provide broad parameters for PGT use, such as to ‘select against genetic conditions, diseases or abnormalities that would severely limit the quality of life of the person who would be born’ (but prohibit selection in favour of a genetic condition).^6^ They do not list a series of genetic conditions, diseases or abnormalities that would satisfy these requirements. However, they offer a series of considerations for professionals (eg, evidence about the impact of the condition, disease or abnormality and concerns of the intended parents about the ability to care for a person born with such a condition). These guidelines, in jurisdictions where they apply, in turn, permit clinicians and providers to assess the ethical acceptability of PGT on a case-by-case basis. Such an approach is premised on the fact that providing an exhaustive list of conditions is impossible. Instead, the guidelines acknowledge the fact that the use of PGT requires ‘serious ethical consideration’ and needs to account for the plethora of community views about the quality of life of persons who may be born with such conditions, diseases and abnormalities (and the fact that such views have the potential to evolve, particularly as new treatments become available), in addition to any potential risks of stigmatisation and discrimination.^48^ ^7^

Other jurisdictions have adopted a ‘medical-indication model’ whereby permitted PGT indications are prescribed.^43^ One example of this model is in the UK, whereby PGT is permitted in cases where there is ‘a particular risk that the embryo to be tested may have a genetic, mitochondrial or chromosomal abnormality, and the Authority is satisfied that a person with the abnormality will have or develop a serious disability, illness or medical condition’.^49^ ^8^ The specific conditions that satisfy these criteria are largely prescribed. For newer indications, the fertility clinic will need to submit an application to the Human Fertilisation and Embryology Authority (hereafter referred to as the ‘HFEA’, which is an oversight body that oversees the licensing and monitoring of clinics), who will then decide whether to add the condition to the list, taking into account a range of factors.^50^

Another regulatory model is the ‘individual requests model’, which involves utilising a regulatory body or ethics committee to approve each case. For instance, in Germany, PGT is permitted if one of the parents has a severe hereditary genetic condition that can be passed on or if there is a high risk of stillbirth or miscarriage.^43^ An independent ethics commission decides the outcome of each request on a case-by-case basis. Each case considered (regardless of whether approval was granted) is documented in a national registry.^43^Despite being able to be approved on a case-by-case basis, Siermann *et al* have suggested that the current regulatory framework does not permit PGT for polygenic conditions.^43^

### Considerations for future regulation

Having briefly outlined some high-level approaches to regulating PGT (noting that the examples above do not currently extend to PRSs but could be adapted to do so), this section now draws on these models as well as other regulatory considerations to offer some regulatory options to inform policymakers (see table 1 for a summary of considerations). Where relevant, some of the ethical considerations canvassed earlier in the article will also be drawn on in light of such options.

**Table 1:** Overview of some relevant regulatory considerations

	Examples of considerations^*^	Potential options (with combinations possible)
**Regulators**	Who should regulate the use of PRSs?	State Industry/commercial entities Profession Through professional organisations Through profession representation within a regulatory body Dedicated oversight body^†^
**Regulatory tools**	What regulatory tool(s) are relevant and/or should be used?	Law Legislation ± associated delegated instrument that can be used to prescribe additional regulations/guidelines Case law Policy/guidelines/standards (variously described)‡ Education These regulatory instruments could explicitly target PRSs in the reproductive context or operate more broadly (eg, targeting assisted reproductive technology generally or drawing on broader relevant regulatory frameworks such as those pertaining to quality and safety or consumer protections).
	How should the regulatory tools enforce their obligations (or exert coercive force)?	Inbuilt sanction for non-compliance such as: Imprisonment Fines (to individual or entity) Finding of professional misconduct (and associated sanctions) Make compliance a prerequisite for: Funding (eg, through links to accreditation of an entity) Individual credentialling (or maintenance of credentials)
**Eligibility criteria**	When should PRSs be available if they are deemed permissible in the reproductive context?	During preimplantation embryo screening supplementary PGT-PRS non-supplementary PGT-PRS
	What traits should PRSs be available for?	Disease Familial conditions that meet the defined threshold Any disease that meets the defined threshold Non-disease traits
	How should permissible traits be identified?	Pre-determined set-list Case-by-case basis Determined by providers/clinicians taking into account broad brush considerations/threshold Require approval by an external body in each case
**Information requirements and education**	How should informed consent requirements be prescribed?	Reliance on existing informed consent principles Specific informed consent requirements (with or without dedicated sanctions for non-compliance)
	Whose responsibility should it be to provide the consumer with the necessary information?	Dedicated health professionals specifically trained to deliver the information (eg, genetic counsellors) Specifically credentialed professionals Any appropriate health professional Designated impartial information service not directly affiliated with any clinic offering the service
	What information should be provided to consumers?	Risks and benefits of the technology Any conflicts or commercial interests of the individual or entity offering the service Nature of PRSs and how they are calculated Including limitations of PRSs such as: the probabilistic nature of PRSs the fact the PRSs can only provide information about relative risk rather than absolute risk the sensitivity and specificity of the technology the influence of environmental and sociodemographic factors (sex, age, ethnicity)
**Ongoing research, development and consultation**	What else needs to be done before (and following) implementation?	Continued efforts to improve current databases (including diversifying cohorts, testing for more diseases and standardising formulas) Continued efforts to improve the technology Further research into: the technology used to calculate PRSs the perceptions, attitudes and decision-making processes of potential consumers (including Collective Reflective Equilibrium) Continued stakeholder consultation regarding social, legal and ethical implications of the technology, which can also be used to help inform future frameworks and policies (or revision of current ones)

* We offer some potentially relevant considerations as a starting point for jurisdictions, we do not purport that these considerations are exhaustive.

† For completeness, we note that the oversight body could be operated by the state (or on behalf of the state) or could operate independently of it.

‡ We note that nomenclature relating to various regulatory tools varies across different contexts, and different jurisdictions assign slightly different meanings to some of these regulatory instruments (eg, policy, guidance, professional guidance and standards) and the coercive force of these instruments vary. Broadly, our reference here is to refer to documents which are not law and that aim to prescribe conduct (originating either from the profession itself, the state or dedicated regulatory body) and/or documents which aim to distil principles or guidance which reflect ‘best practice’ often informed by evidence or professional norms.

PGT, preimplantation genetic testing; PRSs, polygenic risk scores.

#### Regulators and their regulatory tools

A threshold issue to consider is who will be the regulator and what regulatory tools they will employ. Modern conceptions of regulation recognise its decentralised nature and that regulation is not merely achieved through command and control methods (ie, law issued by the state) but can also occur by non-state actors and by regulatory tools other than law (eg, policy, guidelines and education).^51^ In the reproductive medicine context, regulation has occurred through state actors and non-state actors (eg, self-regulation by the profession) or a combination of both. Similarly, both hard and soft forms (ie, non-legally binding) of regulatory tools have been used in this context and are often used concurrently and employed in a mutually reinforcing manner.^44^

While there is no consensus about who is best placed to regulate assisted reproductive technologies, there are compelling arguments for an ever-evolving field such as reproductive medicine to rely on the profession to regulate either solely (or in conjunction) with the state. The less prescriptive the state is with respect to its parameters of PGT, the more discretion the profession has to regulate. The advantage of the profession playing a role in regulating PGT is that its members are more likely to be acquainted with patient needs, technological limitations, new developments and the day-to-day challenges of clinical practice.^46^ However, regulation exclusively by the profession can be problematic, particularly in cases where professional organisations with a profession-specific membership take primary responsibility for regulation. This is because such organisations are ultimately there to represent and protect their members (rather than society more broadly). Therefore, any form of regulation likely to make their work more difficult (particularly if this correlates with increased administrative responsibilities) could lack appeal, even if it is the most appropriate means of minimising harm to consumers.^52^

A middle ground between exclusively state regulation and solely self-regulation can be achieved when bodies develop professional guidelines. For instance, the HFEA and NHMRC, who are responsible for developing guidelines in the UK and Australia, respectively, are statutory bodies but have a degree of discretion when it comes to developing guidelines (within broad state parameters). Each of these bodies has representation from members of the profession but not exclusively. Indeed, they also have representation from other stakeholder groups such as consumer advocates, legal representation and Government ministers. A diverse membership ensures that the profession has the means to shape regulation but does not have a monopoly.

Further, even if the profession has a greater role in day-to-day regulation than the state, the state can still aim to regulate this practice, even if it is done indirectly. For instance, the state could still be permitted to regulate indirectly through quality and safety standards (for the technology used), consumer protections and advertising standards (eg, for controlling how the technology is marketed to the consumer). This could be particularly useful in cases where DTC companies offer PES, as it is unclear whether industry leaders are likely to adopt recommendations from health professional bodies if they are not binding.^53^

It is also essential to consider the regulatory tools that regulators rely on. The coercive force of law has some appeal, but it is often difficult to reform and consequently lags behind scientific innovation. Softer forms of regulation provide a means to more readily respond to scientific advancements without undertaking the lengthy law reform process. While this may be preferable to state regulation (particularly in the context of inflexible or poorly drafted laws), issues arise when professional guidance leaves open broad permissibility (subject to overarching ethical and professional obligations) and invites clinicians’ discretion in relation to what services to offer, particularly in cases where there could be a perceived conflict of interest. There is also a risk that guidelines may not reflect the broader community’s views and expectations. However, community consultation could mitigate this.

Another caveat with guidelines is that consequences of non-compliance are often perceived as less severe than law. This is particularly so in cases where professional organisations solely issue guidelines. This is because, in such cases, there is heavy reliance on voluntary compliance and scrutiny of peers to regulate behaviour rather than close monitoring (as evident in the current US approach). However, as currently occurs in Australia and the UK, guidelines can be linked with accreditation whereby compliance is a prerequisite to clinics being accredited (which may be mandatory for clinics to operate or receive funding), which can serve as a coercive force and motivate compliance.

Alternatively (or additionally), the state could be charged with primary regulatory responsibility but embed flexibility within its regulatory approach. This could be achieved, for example, by having mandatory periodic reviews. Flexibility could also be achieved through using delegated forms of regulation^9^ in jurisdictions where this is permissible. In such cases, the delegated instrument (eg, subordinate legislation or guidelines) could be issued by a single state representative (eg, government minister) rather than having to go through a parliamentary process. Such an approach could be useful in relation to aspects of the regime that may need to change in response to technological developments and/or societal views (eg, eligibility).

#### Availability and eligibility for access

In addition to decisions being made about who will regulate and through what means, any jurisdiction considering using PRSs in the context of reproduction needs to make a threshold decision as to whether they wish to use this technology, which may be informed by current evidence and consultation (as discussed in more depth below). In making such decisions, adopting a parity principle may assist policymakers in looking to similar technologies currently used to see if PRSs meet a similar epistemic bar.

If it is decided that this technology should be introduced, then jurisdictions need to make decisions in relation to the eligibility and availability of this technology. Accordingly, consideration needs to be given as to whether only what we defined earlier as ‘supplementary PGT-PRS’ (people who would otherwise be eligible for IVF calculate PRSs to select embryos) or whether ‘non-supplementary PGT-PRS’ (people access IVF for the mere purpose of calculating PRSs to choose an embryo^10^) should be permitted too. In making such decisions, the harms associated with both forms of PRSs (such as those previously discussed) should be considered in addition to potential interventions or policies to address such harms. This could be achieved, for example, by introducing minimum information requirements and education to mitigate decision-making difficulties (discussed further below), mandating parental counselling to address parental expectations or introducing policies around managing any excess embryos generated.

Consideration should also be given to whether this technology should only be for disease traits or extend to non-disease traits and how many traits should be permitted to be tested simultaneously. In cases where conditions are limited to disease traits, consideration needs to be given to whether there should be a predetermined list of conditions/traits set out by the state (similar to how the UK currently regulates PGT) or whether the use of PGT is determined on a case-by-case basis using a broad set of criteria either by providers/clinicians (similar to the approach of most Australian jurisdictions that rely on NHMRC guidelines) or by a dedicated committee (similar to Germany’s current approach). Consideration should also be given to whether PRSs can only be calculated based on diseases in which there is a familial history. For instance, Chin *et al* suggested that PRSs in the context of embryonic screening should only be available for diseases diagnosed in the embryo’s parents or grandparents, and prospective parents should provide evidence to this effect.^23^ This would mean that while multiple conditions could be tested simultaneously, parents would only be permitted to calculate a PRS for familial conditions. This, in turn, could reduce some of the decision-making burdens discussed previously. Consideration could also be given to whether adult-onset polygenic diseases should be treated differently than those present earlier in life, given the potential for environmental factors to influence the phenotype.

If this technology is used in the context of non-disease traits,^11^ similar considerations also arise regarding whether there should be a predetermined list of permissible non-disease traits or whether they should be offered on a case-by-case basis (determined by a clinician or a committee). However, consideration would also need to be given to what will be used to define the threshold of permissibility, which is arguably more difficult than determining a threshold for disease traits. One potential model for non-disease traits previously posed by one of the authors is the welfarist model, which allows for the selection of any trait provided it can enhance well-being, which is notably broader than merely one’s health or disease.^37^ If such an approach were permitted, consideration would need to be given as to whether this should be offered on a scalar basis (ie, any increase in well-being will justify use) or whether a particular threshold should be generated (ie, a specific threshold of well-being is necessary).^37^ An obvious caveat with the latter is that it would be challenging to develop an objective measure of well-being and consequently define a threshold.

In making decisions about what traits parents should be able to select, it is important to consider this in light of the eugenic and discrimination concerns identified above. Indeed, while PES represents a more liberal form of eugenics whereby parents are making voluntary decisions to enhance procreative options and the well-being of their offspring as opposed to state-initiated eugenic regimes, any form of ranking of future offspring might support an ideology of legitimising value judgements based on one’s genetic makeup, especially in the case of non-disease traits.^8 35^ Justice concerns related to funding are also relevant in this context as the state needs to decide what types of polygenic screening (ie, supplementary or non-supplementary PGT-PRS) it should subsidise (if any) and which traits.

#### Information requirements and education

In most jurisdictions, PRSs for embryo screening will likely be offered as an ‘add-on’ service for those eligible for IVF in their jurisdiction (at least initially). Hence, consideration must be given regarding the necessary information to facilitate the choice to proceed with this add-on. The need to provide people with enough information to make informed decisions is well entrenched in medical practice and a means of respecting reproductive autonomy. While existing informed consent principles that apply throughout medicine could be relied on, this may invite discretion and inconsistency regarding the level of information provided to individual consumers, with further challenges presenting when offered on a DTC basis (particularly due to competing proprietary interests^54^). Hence, introducing some level of prescription may be beneficial. This added level of prescription is already reflected in much of the current regulation of PGT and artificial reproductive technologies more broadly. For instance, in Australia’s NHMRC guidelines, minimum levels of information must be provided, including options for discarding embryos, whether the treatment or procedure is an accepted or innovative practice, costs associated, competing interests, etc.^48^ However, even when there is some added level of prescription, the matters covered still tend to be framed relatively broadly (rather than reflecting the nuances of PRSs). Hence, further prescription may be needed to discern precisely what should be disclosed in the context of PRSs and what format it should take to minimise variability.

The sheer complexity of PRSs and polygenic diseases creates enormous challenges for communicating these benefits and risks in a way that consumers can fully appreciate and understand, even if they have high health literacy.^18^ While some jurisdictions may have dedicated health professionals, such as genetic counsellors, who are specifically trained to deliver such information, others lack dedicated professionals and may rely on clinicians more generally. In such cases, consideration could be given to introducing mandatory education for personnel offering PRSs in the context of embryonic screening and requiring them to be specifically credentialed to provide this service (and information about it).

When PRSs are offered commercially, particular attention should be given to information provision. Indeed, while these companies will generally offer some form of counselling, they ultimately have commercial interests and benefit if the consumer uses the technology. Hence, the degree to which such personnel can be impartial and transparent about the promise and limitations of this technology should be questioned, particularly given biased information will compromise reproductive autonomy.^2^ Concerns about the degree of comprehensiveness of the information provided to consumers have also been raised outside the commercial context, with some commentators arguing that it is unreasonable to assume, given the complexity of PRSs, that clinic staff will have the time and resources required to convey all the necessary information in a comprehensive, yet accessible manner, to help consumers fully appreciate the risks and limitations needed for informed consent.^42^

Given the aforementioned discussion, consideration should also be given to what information should be provided, who is best placed to give such information, and whether existing information and/or informed consent requirements in relation to reproductive technologies are apt or whether more prescriptive requirements are needed. There is some support for this latter view, with some commentators suggesting, at minimum, patients should be given information about the probabilistic nature of PRSs, the fact that they can only provide information about relative (in contrast to absolute) risk, their limited sensitivity and specificity and how their degree of accuracy is influenced by sociodemographic factors (eg, sex, age and ethnicity) and environment.^9 11^

In terms of who is best placed to provide information, one approach could be to have a dedicated information service established (or repurposed, such as a fertility society) that is then responsible for developing its own resources (including visual aids to clarify information^7^) and who would not have a commercial interest. If consultation with such a body were mandatory, this would ensure that individuals are provided with the necessary information. Moreover, due to the concentrated expertise within the body, the information delivered is likely to be standardised and delivered consistently rather than ad hoc. If this is not possible, requirements that obligate any person providing counselling to disclose potential conflicts should exist, if not already provided for, as part of broader artificial reproductive technology or PGT regulation.^7^

Additionally, consideration should be given to what mechanisms would be appropriate to ensure compliance with such requirements and what sanctions should apply in cases of insufficient information and consequences for providing misleading information (eg, exaggerating the potential benefits deriving from PRSs). Depending on who provides such information, there may be scope to rely on existing obligations relating to informed consent (eg, in the case of health practitioners). Moreover, in cases where such services are offered commercially, consumer law and obligations relating to misleading and deceptive conduct may become relevant. However, if these general mechanisms are not considered apt, for instance, if jurisdictions want to introduce more severe sanctions to encourage compliance or perceive existing mechanisms as difficult to apply in this context (eg, because there may be difficulties arising from quantifying harms associated with decision-making), then consideration of tighter enforcement and stricter penalties may be necessary (eg, imprisonment and fines).

#### Ongoing research, development and consultation

Irrespective of whether the decision is made to use PRSs in the reproductive context or not, there is a need for ongoing research, technology refinement and consultation. The quality of existing databases should be improved, including efforts to diversify current participation in GWAS, test for more diseases and standardise formulas used to calculate PRSs to reduce discrepancies. Improving databases will help inform assessments about this technology’s clinical utility,^16^ including its appropriateness among different cohorts, not just those of European ancestry.

Improving the technology and understanding its utility and limitations also helps ensure reproductive autonomy is respected and upheld to the greatest extent possible. This is because, as previously noted, decisions made on low-quality information have the potential to mislead and, hence, inhibit autonomy rather than promote it. In addition to researching the nature of the technology itself, jurisdictions should also seek to research potential consumers’ perceptions, attitudes and decision-making processes.^8^ This could be used to minimise any potential iatrogenic effects related to the technology but also to help inform guidance documents and approaches to counselling.^8^ Engagement with the public through qualitative and quantitative research could also be used to inform policy through procedures such as Collective Reflective Equilibrium.^12^ ^55^

Diverting more resources towards appropriate research in relation to this technology before widespread implementation may also be justified from a justice perspective, with some commentators arguing that resources would be more appropriately allocated if spent on improving knowledge (eg, how PRSs are influenced by the environment and pleiotropy).^9^ It has also been suggested that commercial companies who want to use this technology should do this via research protocols at no extra cost to generate further evidence about the potential effects of the technology.^11^

However, these considerations must be carefully weighed against other research projects competing for scarce funding and resources. To the extent that substantial public funding is to be devoted to improving PRSs for embryo selection, there must not only be a clear public value proposition but one that is more substantial than other areas where those resources could be spent. These determinations may be informed by consideration of the significance of values of reproductive autonomy (qua providing prospective parents with more reliable information) and harm prevention (qua reducing the prevalence of harmful conditions), which could be advanced through more reliable PRSs.

Stakeholder consultation is also needed regarding the social, legal and ethical implications of PRSs in the reproductive context to aid integration into practice and for the development of the appropriate regulatory tools and supporting resources.^7^ Consultation should also inform ethical frameworks underpinning policies. Such frameworks should capture, *inter alia*, any potential harms and the relevant values and priorities of the community to which they apply. In this way, consultation can help inform contextually sensitive answers to regulatory challenges, such as the extent to which individual liberties should be constrained to protect patients. Consultation should be ongoing so that learnings from practice can be integrated to improve practice over time and assist in optimising regulation. It should draw on the insights from various stakeholders, including patient advocates, ethicists, scientists and health professionals (including representation from professional bodies).^7^

## Conclusion

Using PRSs for embryo screening presents several opportunities but raises several ethical and regulatory challenges. While the use of PRSs is currently limited in the reproductive context, further refinement of genetic technology and the genetic databases used to generate PRSs may see their use in the reproductive context expand. Outside of jurisdictions that take a laissez-faire approach to regulating the use of reproductive technology, many relevant regulatory frameworks in their current form are arguably not apt to regulate the use of PRSs. Therefore, further consideration about regulating this new practice before it becomes more widespread is necessary. Regulatory considerations should extend beyond the normative question of whether such a practice should be implemented and instead broaden the focus to account for other regulatory considerations such as appropriate regulators and regulatory tools, eligibility criteria, information and education requirements, and the need for further (and ongoing) technology refinement, research and consultation.

## Data Availability

No data are available.

## Footnotes

Specificity refers to the proportion of people who were designated as low risk and did not develop a condition. Sensitivity refers to the proportion of people who were designated as high risk and did develop the condition.

For completeness, we note that there have been efforts to address this, with some attempts to try and diversify participants in such studies to enhance their generalisability and overall utility.

Embryo Selection based on Polygenic Scores (ESPS) and PRS for Embryo Selection (PRS-ES) have also been used to describe polygenic embryo screening.

While our main focus is on embryo selection by prospective parents, we note that other personnel such as embryologists may also wish to rely on such information for the purposes of morphology evaluation.

It is also worth noting that in the case of supplementary PGT-PRS the non-identity issue arises, whereby harm cannot be said to occur to the future child because choices are determinative of existence. Therefore, supplementary PGT-PRS cannot be said to harm the individual in the counterfactual sense. We note this for completeness but do not have the scope in this article for richer engagement.

For completeness, we note PGT is also possible to ’select an embryo with compatible tissue for subsequent stem cell therapy’ and ‘increase the likelihood of a live birth’.

This approach arguably invites a degree of flexibility and discretion; however, despite leaving the door open for interpretation, it is unlikely this extends to the context of PRSs. Indeed, recent amendments in 2023 to the Guidelines saw a revision of terminology to define PGT which explicitly mentioned PGT-A, PGT-S and PGT-M, with mention of PGT-P noticeably absent.

Medical sex selection is also permitted.

Delegated or subordinate legislation is a form of legislation that is issued by a state actor (eg, a responsible minister) based on powers conferred to them by the state, rather than a piece of legislation passed by Parliament (often referred to as primary legislation). We note, however, the possibility of this will be confined to those jurisdictions that permit subordinate instruments.

For completeness, we note, that there is arguably a moral difference in the case of non-supplementary PGT and whether it is used to select the ‘best embryo’ or select against a specific familial disease, which we subsequently address.

We note that there are differences between disease and non-disease traits, which have been subject to much debate. There is no scope within this paper to discuss such differences at any length other than canvass a particular threshold.

Collective reflective equilibrium involves bringing ethical concepts, theories and principles into maximum coherence with public intuitions to form policy, so they have greater justification and legitimacy.
